# Mutual Relations of Physician and Layman1President’s address delivered at the Eighty-ninth Annual Meeting of the Medical Society of the County of Erie, December 19, 1910.

**Published:** 1911-02

**Authors:** Grover W. Wende

**Affiliations:** Buffalo, N. Y. 471 Delaware Avenue


### BUFFALO MEDICAL JOURNAL

VOL. LXVI. FEBRUARY, 1911. No. 7

### ORIGINAL COMMUNICATIONS

### Mutual Relations of Physician and Layman[1]

BY GROVER W. WENDE, M.D.
Buffalo, N. Y.

THE society closes the eighty-ninth year of its existence in circumstances upon which its presiding officer congratulates its members. In progress toward professional unity, as well as numerical strength, its development has been more than satisfactory. At no time in the history of this organisation has its exalted purpose to benefit its members and the public been more faithfully carried out, or with greater promise. Every effort in this direction has met with such hearty coöperation that the profession, without regard to sectarian bias, has come to recognise the value and influence of this society.

We are now entering upon a new era which is characterised by higher professional ideals and by determined effort toward the substitution of scientific treatment for empiricism in all branches of medical practice. It should be remembered that the environment of the medical profession is the same that has always existed; the obstacles, while numerous, are not new.

If they are somewhat modified their nature remains much the same, and their influence is in no sense weakened. The credulity of the public has availed to crowd the world with medical pretenders. Deny it as he may, the medical practitioner is forced to compete with them, not only for a livelihood but, far more important, for the welfare of the human race.

IMPORTANCE OF ORGANISATION.

This society, as a factor in the effort to unite the entire profession of the state and the nation, has a higher and nobler object than its own immediate advantage. Only by the combined efforts of kindred organisations can the future health and welfare of society be conserved. As a profession we are dedicated to the service of humanity. This service can no longer be effectively rendered individually but only collectively by complete organisation. This organisation demands the coöperation of each and

---

1. President's address delivered at the Eighty-ninth Annual Meeting of the Medical Society of the County of Erie, December 19, 1910.

every individual member of our profession welded together into local, state, and national bodies, into a great organised army equipped for united effective service. No physician can perform his full duty and remain apart from such organisation. We will welcome into our ranks all worthy physicians who are still outside.

RELATION OF PHYSICIAN AND LAYMAN.

One of the saddest facts connected with our social life is the strained and awkward relation existing between the physician and the layman. The honest, painstaking and self-sacrificing members of our profession are in too many instances regarded as the enemies rather than the sympathetic and benevolent friends of the people. Their benevolence is not infrequently translated into selfishness. Their integrity is doubted, their motives are impugned, their character is assailed and their reputation suffers through the prejudices and caprices of those who really need them most.

The want of legitimate confidence in the physician upon the part of the layman is mainly provocative of the existing condition. And this lack may be traced to the amazing prevalence of empiricism. For some unexplained reason, the quack is able to gain confidence where it is denied to the regular practitioner. Whether this is due to the accepted fact that human nature seems to relish humbug, to enjoy the very deception by which it is cajoled, or, is due to weakness, carelessness, or caprice, remains an open question. It becomes something to wonder at when laymen calmly discount the efficient culture of the scholar and welcome to their confidence, their homes and the bedsides of their sick, any arrogant pretender whose advent is proclaimed by fulsome, extravagant advertising and vulgar display. Sheer worthlessness is thus brought into active competition with genuine merit, pretence with culture, and doubtful and dangerous methods and remedies with the established ideals and requirements of a conscientious, broad and educated profession.

This explains why, in so many instances, sanitary reforms are hindered or prevented, and institutions projected in the interest of the general health are rendered so difficult of establishment. Small politicians are always eager to take advantage of the state of things here defined, and in them graft finds encouragement for its rapacious exercise. So one bad thing leads to another. The progeny of unwarranted things is proverbially prolific. Like begets like in evil as well as good.

The fundamental fault of the layman, in his relation to the medical profession, is his failure to give proper consideration to the just claims of that profession, to appreciate its sincerity, to recognise its mission and to credit and confide in its worthy individuals. Until the true physician is appreciated and differentiated from the pretenders now flourishing by the credulity and gullibility of the public, he will continue to suffer as he suffers now. Money getting is far from being the primary and ruling consideration with the conscientious physician. Where human suffering is involved, where life itself is at stake, he would far rather give than get; would rather sacrifice than aggrandize. There are few who dishonor the profession by acting otherwise. The genuine physician is moved by an impulse truly altruistic. He is inspired by a lofty ideal. The sentiment that invests his profession becomes his own. Trusted and confided in and coöperated with, instead of suspected, misrepresented and held at a discount, he becomes both friend and benefactor. However they may seek to avoid the issue, however reluctant they may be to admit what is here claimed, the fault in this matter is confined to the laymen themselves. They are responsible for the evil and will be held responsible for its continuance.

It remains to be said that this evil can be greatly modified by discreet and proper legislation; by the adoption of measures designed to suppress quackery; and by educating the public to an appreciation of the altruistic purpose of medical societies. Logically, this is the initial step in the right direction. When public attention is narrowed to the profession itself, its claims will come to be more fairly considered, and a recognition of its virtues will be an easy sequence. Ignorance, caprice, stubbornness, all play a part in this deplorable matter. There is no logical or weighty reason why the relation between medical practitioner and layman should be strained or unfriendly, or why the motives of the former should be misunderstood. Let the layman exercise common sense in considering the nature, characteristics, claims and sentiment of the medical profession; let him recognise its culture, experience and sympathetic service; let him believe in the honesty of motive and practice of its members,—in a word, let him be fair-minded and justly discriminating, and the desired result will be attained. When laymen, instead of eagerly and blindly taking the first thing that may happen along and allowing themselves to be deceived by impositions; when they pause, think, reason and consider, their own sober sense will, in the end, create in them a better mood and lead them to saner and safer decisions.

Public credulity once gone, the flock of empirical buzzards must straightway seek their carrion in other fields. It therefore becomes the duty of the medical profession to aim its most determined aggression directly against this widespread evil and in this effort again the powerful influence of the united profession through our societies would be effective.

POPULAR MAGAZINES AND MEDICINE.

It is a significant sign of the times that the best and most influential of our popular magazines have formed the commendable habit of discussing medical subjects, a fact that has resulted in more general and appreciative knowledge concerning the profession and has assisted in producing a higher estimate of scientific research and result upon the part of the general public. The number of readers of these articles is gradually increasing and will include the less educated and more prejudiced. Not only is laudable curiosity being satisfied but erroneous and superstitious notions are being abandoned, while a more intelligent understanding of pathological conditions and of public hygiene is being secured.

The time will arrive when laymen will permit medical men to speak for themselves, instead of insisting upon speaking for them; when laymen will be willing to admit that educated physicians know more than they about medical matters, and when the opinions of expert physicians will be sought and followed, even by politicians, in questions of public hygiene, sites for hospitals and their construction, pure water supply, health of school children, and similar subjects,—matters of supreme importance to the public health and welfare.

CONTAGIOUS DISEASES HOSPITAL.

It may be cited by way of illustration of past and present methods that three years ago a committee was appointed by this society to consider the important question of the proper location and construction of a municipal hospital for contagious diseases. The committee was composed of men worthy to be ranked as experts on the subject, and their reputation and exhaustive labors entitle them to general confidence. With unselfish devotion and fidelity to the trust imposed in them, they exhaustively investigated the subject in foreign countries and in many places in our own country and reported their findings and recommendations. In spite of this, politicians by means of various misrepresentations and appeals to false local prejudices negatived the labors of the committee and prevented the acceptance of their recommendations. As a result of the confusion thus produced, by which even educated men became influenced, the humane project was defeated or at least delayed. Opprobious terms, such as "pest house" and "danger hospital," in the editorials of newspapers were applied with the idea of bringing the project into disrepute, while the safety of the public continued to be jeopardised through ignorance and greed. Even a clergyman, presiding over one of the largest congregations in Buffalo, proclaimed at a public hearing, and it is known that a large percentage of his followers accepted the statement, that he knew as much about contagious diseases as the best of doctors, a statement that would be amusing provided it affected himself alone.

In happy contrast to such ignorance, indifference and hostility, emanating from laymen and lay bodies is the attitude of confidence usually shown by responsible municipal, state, and national departments of charity and health, in welcoming the coöperation and critical investigations by organized medical societies of all kinds of measures proposed for the improvement of the administration and control of sanitation and hygiene. Illustrating this proper spirit of respect toward the scientific and organised medical profession may be cited the receipt by the president of this society of a recent communication from Health Commissoner Fronczak, expressing his sincere appreciation of the interest and coöperation of the Medical Society of the County of Erie in helping the Department of Health in the correct solution of all debatable questions of administration, reforms and progress relating to his department. He asks the further support of this society in helping to improve the quality and efficiency of medical officers upon whom rests the responsibility of guarding the public health through medical, public school, food and drug inspection, and by all other means of sanitary control.

### THE MEDICAL PRACTICE ACT.

It redounds to the credit of the Medical Society of the County of Erie that due to the untiring efforts of one of its members, Dr. H. R. Hopkins, the state of New York instituted the public control of the membership of our profession by the adoption of the medical practice act, a statute that has served as a model for many other states. Dr. Hopkins's active service to the state is fortunately not closed, but is being pushed still farther in his commendable effort to secure the adoption by the state of a materially advanced standard of qualification for admission to the study and practice of medicine. The advisability of the adoption of a standard of qualification for medical practice is also carefully considered in the report of our special committee on the "Division of Fees," of which Dr. John H. Pryor is chairman. The recommendations therein suggested are entitled to our most deliberate and careful consideration.

I now desire to suggest that such new committees as may be appointed shall include many of the younger and newer members who have not heretofore been represented in special committee assignments, to the end of securing an infusion of new blood and vitality and of diffusing the spirit of active interest and participation in our important organised work.

### POPULAR LECTURES BY PHYSICIANS.

At the 1909 meeting of the American Medical Association, it was recommended that the work of public education in hygiene be undertaken by the women physician members of the association, by means of public lectures upon relative topics. This matter was presented early in 1910 to this society but nothing has resulted. The chair would suggest that the consideration of this important subject be resumed, with a view to definite action, for the benefit of the laity. This plan includes popular lectures by various members upon subjects calculated to educate a class too long denied the information it so genuinely needs, correcting various popular delusions and doing away with many causes of unwarranted fear.

### UNION OF COUNTY SOCIETY AND ACADEMY OF MEDICINE ADVISED.

At present there exists in Buffalo two general medical societies,—the Medical Society of the County of Erie and the Buffalo Academy of Medicine. These two societies are in the matter of membership, aim of purpose, spirit of fraternity, and non-sectarianship practically identical. They are separate and distinct only in name, organisation, administration, and time and place of assembly. They perform the same general functions. They duplicate each other without special purpose. Historically, their separate existence was justified. Today their separation only weakens both. Is there any important reason for their continued separation? Would not their consolidation be mutually advantageous? It is recommended that suitable action be initiated on the part of this society looking to proposals of consolidation with the Buffalo Academy of Medicine. If technical barriers to such union be found to exist, surely a spirit of mutual goodwill can be trusted to overcome them.

### THE COUNCIL.

During the past year the Council has met fifteen times and as president I wish to acknowledge my indebtedness to its members for the creditable attendance at meetings; hearty coöperation and earnest assistance in considering and disposing of the mass of material coming before it for action; all matters which were to be presented to the society were first carefully investigated by this Council, in order that nothing so presented should lack mature and careful preliminary consideration. The wisdom of referring all matters to come before the society to the council before presentation has been apparent in the easy and rapid disposal of the great amount of business coming before the society for its action. The society should heartily appreciate the amount of time, thought and discussion given to its affairs by this executive body.

### BOARD OF CENSORS.

The Board of Censors, of which Dr. John H. Grant is the efficient chairman, has prosecuted its difficult labors with an energy that deserves hearty commendation and with a success that is notable. The chairman has presented a report of the board's actions at each meeting and will present his final report tonight. His devotion to duty and sacrifice of time to the prosecution of the arduous police duties of the society demand an adequate expression of appreciation by our membership.

### COMMITTEE ON PUBLIC HEALTH.

The many questions coming before the Committee of Public Health will be fully presented by its able and untiring chairman, Dr. Henry R. Hopkins, and I ask your careful consideration of the important suggestions that will be offered. Without actually knowing the complex conditions from which the information for this report was obtained, you cannot realise the amount of time and labor devoted to its preparation.

### COMMITTEE ON LEGISLATION.

The Committee on Legislation, under its able chairman, Dr. F. Park Lewis, has been active in supporting legislative reforms in the state. The fact that all matters relating to legislation must pass finally through the state committee has operated to diminish the personal credit due our excellent committee. I also ask your careful consideration of the suggestions to be presented by this committee.

### COMMITTEE ON MEMBERSHIP.

The president desires to make special acknowledgment of the faithful and excellent work of Dr. Thomas H. McKee, the chairman of the membership and entertainment committees. It is gratifying that the increase of membership during the present year has never been equaled. It is believed that this increase exceeds that of any other county society in the state, that of New York included. The chairman of the membership committee has always safeguarded the quality of our membership and only deserving applicants have gained entrance to the society.

### MILK COMMISSION.

Under the provisions of a law of the state of New York the chair appointed a milk commission whose duty is to regularly investigate and certify to such dairies as comply with the legal requirements. The president of the milk commission is Dr. C. Sumner Jones, who has devoted much time in investigating the dairies desiring certification; his efforts have been crowned with success as will be shown by his report to be presented later.

HOSPITAL FOR CONTAGIOUS DISEASES.

Some three years ago a special committee was appointed by this society to secure a municipal hospital for the care of contagious diseases, under the chairmanship of Dr. John D. Bonnar. The work of this committee has been patiently and efficiently done, the chairman himself exhibiting an energy and a faithfulness beyond all praise. That the desired result is still delayed casts no reflection upon either the committee or its indomitable chairman.

COMMITTEE OF INVITATION TO A. M. A.

The chair appointed a special committee to which was assigned the duty of inviting the American Medical Association to hold its 1911 meeting in Buffalo. The chairman of the committee, Dr. Charles G. Stockton, handled this difficult matter in a manner entitling him to all praise, and he was ably supported by the allied members. An exhaustive folder was prepared by Secretary Lytle which should have exerted great influence. It is creditable to the committee and to the society that its efforts in the convention were free from the taint of Association politics and that only clean and open methods were adopted in the hope of securing a favorable result. In the final result only two votes were lacking to reward the efforts of the committee with success.

DIVISION OF FEES COMMITTEE.

Grateful mention deserves to be made of the work of the special committee, Dr. John H. Pryor, chairman, appointed to investigate the subject of the division of fees. This committee was appointed in response to the unanimous request of the society, expressed by a rising vote. The bold and honest stand taken by the committee in its report should receive the approbation of the society, as it will be heartily endorsed by every worthy member of the profession throughout the land.

THE SECRETARY AND THE TREASURER.

Without specifying the remaining committees, it is only fair to say that their efforts have all been earnest and then work excellent. The worthy secretary of the society, Dr. Franklin C. Gram, could not be praised in adequate terms, even if superlatives alone were used. He has proved himself not only the president's right-hand man, but a man always right on hand. His duties have been pressing and his labors arduous, but there has been no flinching or failing. He deserves and should receive the grateful thanks of us all. As for the treasurer, Dr. Albert T. Lytle, he has accomplished for the society what none of his predecessors ever even attempted. His methods have been original and his efforts untiring, while to his amiable persistence in the case of delinquents, many of you can bear witness. His faithful service rightly entitles him to the best we can give him in return. It may be said right here, that presidents come and go, which is, perhaps, a favorable provision, but secretaries and treasurers such as those of this society, like Tennyson's brook, should "go on forever."

AN APPRECIATION.

I desire, finally, to express my full appreciation of the distinguished honor conferred upon me by this society in choosing me as its president, and my heartiest thanks to all the members for their loyalty and support during the year. If my service has not been all that you or I would have had it, it has been supported by my highest purpose and my utmost ability. Although another might have served you more efficiently, he could not have done it more willingly. My most cordial regards to you all and my best wishes for every member.

471 Delaware Avenue.

